# Financial Outcomes Associated With the COVID-19 Pandemic in California Hospitals

**DOI:** 10.1001/jamahealthforum.2022.3056

**Published:** 2022-09-23

**Authors:** Yu Wang, Allison E. Witman, David D. Cho, Ethan D. Watson

**Affiliations:** 1 Congdon School of Supply Chain, Business Analytics, and Information Systems, University of North Carolina, Wilmington; 2 Department of Economics and Finance, University of North Carolina, Wilmington; 3 Department of Management, College of Business and Economics, California State University, Fullerton

## Abstract

**Importance:**

The COVID-19 pandemic challenged the financial solvency of hospitals, yet there is limited evidence examining hospital financial performance through the first 15 months of the pandemic.

**Objective:**

To assess the financial outcomes associated with the COVID-19 pandemic in California hospitals.

**Design, Setting, and Participants:**

This cross-sectional study tracked the financial performance of 348 hospitals in California using Hospital Quarterly Financial and Utilization Data from the State of California Office of Statewide Health Planning and Development. Hospital financial performance was examined from January 2019 to June 2021 for all hospitals in aggregate and by safety-net status.

**Exposures:**

Pre–COVID-19 financial outcomes vs COVID-19 period outcomes.

**Main Outcomes and Measures:**

Quarterly revenues, expenses, and profits.

**Results:**

In 348 California hospitals, hospital financial performance was highly variable during the COVID-19 pandemic. Losses were reduced by COVID-19 relief funding and strong equities market performance starting in the second quarter of 2020. Non–safety net hospitals maintained positive operating margins throughout the pandemic, while safety-net hospitals experienced large losses. Between the first quarter of 2020 and the second quarter of 2021, California safety-net hospitals’ net operating losses were more than $3.2 billion.

**Conclusions and Relevance:**

In this cross-sectional study of California hospitals, hospital financial performance was tracked between the first quarter of 2019 and the second quarter of 2021. Although hospitals experienced reduced profits between January 2020 and June 2021, the interventions of government assistance programs were able to mitigate more detrimental fiscal consequences. When compared with non–safety net hospitals, safety-net hospitals were confronted with more concentrated financial losses.

## Key Points

**Question:**

What are the financial outcomes associated with the COVID-19 pandemic in California hospitals?

**Findings:**

In this cross-sectional study of 348 California hospitals between the first quarter of 2019 and the second quarter of 2021, hospital financial performance was highly variable during the COVID-19 pandemic. Financial losses were reduced by COVID-19 relief funding and strong equities market performance starting in the second quarter of 2020; losses were concentrated among safety-net hospitals, with operating losses totaling more than $3.2 billion across 101 California safety-net hospitals between the first quarter of 2020 and the second quarter of 2021.

**Meaning:**

In this study, government assistance programs were found to play a substantial role in lessening the financial damage of COVID-19 for hospitals in California, especially those with safety-net status.

## Teaser

This cross-sectional study uses quarterly data from 348 California acute care facilities between 2019 and June 2021 to assess financial outcomes, government financial assistance programs, and safety-net hospital performance compared with non–safety net hospital performance.

## Introduction

The COVID-19 pandemic placed substantial pressure on the US health care industry and caused substantial disruptions to the acute care hospital system. Beginning in March 2020, many hospitals cancelled elective and outpatient procedures to accommodate potential surges of COVID-19 in patients and reduce hospital transmission of the virus. Elective and outpatient procedures are important factors in a hospital’s financial health, accounting for approximately 63% of an average hospital’s revenue in 2018^1^ and their cancellation posed serious challenges to the financial solvency of many hospitals.^2,3^ Although increased admissions for patients with COVID-19 might have partially offset lost revenue from other procedures, the American Hospital Association estimated a total loss of $202.6 billion by American hospitals between March and June 2020.^4^

Realizing the potential destabilizing financial consequences of COVID-19 for health care professionals and facilities,^5,6^ the US federal government enacted a number of assistance programs. Beginning in April 2020, the Provider Relief Fund (PRF) granted hospitals participating in Medicare 2% of their previous year’s patient revenue.^7^ Subsequent PRF distributions were targeted at rural hospitals, safety-net hospitals (SNHs), and health care institutions serving a high share of Medicaid patients. Other financial assistance came in forms such as a 20% increase in Medicare payments for patients with COVID-19, loans through the Medicare Accelerated and Advance Payment Programs and the Paycheck Protection Program, state-funded increases in Medicaid payments in many states, and reimbursements to health care organizations for testing, treatment, and vaccination of uninsured patients.^8^ The scale of financial assistance given to hospitals was unprecedented and helped the overall profit margins of hospitals in 2020 to remain similar to prior years, especially for government, rural, and smaller hospitals.^9^

Similar to government, rural, and smaller hospitals, SNHs traditionally operated on thinner margins prior to the pandemic^10^ and might have been disproportionately disrupted by COVID-19 both financially and operationally.^11,12^ Consequently, the patient populations served by these SNHs were vulnerable to reduced access to health care services. To address this situation, the PRF contained a $10 billion targeted distribution for SNHs.^13^

This cross-sectional study analyzed the financial performance of California hospitals overall and by SNH during the first 6 quarters of the COVID-19 pandemic. Observing continuous dynamics of hospital financial performance is important to an understanding of how hospitals fared as cases rapidly surged then receded multiple times in 2020 and 2021. Using quarterly data from 348 California acute care facilities between 2019 and June 2021, this study focused on 3 research questions: (1) What happened to California hospitals financially during the first 18 months of the COVID-19 outbreak? (2) What roles did government financial assistance programs play? and (3) How did SNHs fare compared with non-SNHs?

## Methods

### Data Collection

We used Hospital Quarterly Financial and Utilization Data from the State of California Office of Statewide Health Planning and Development.^14^ The University of North Carolina Wilmington Office of Sponsored Programs and Research Compliance reviewed this study and determined that this study did not meet the regulatory definition of human participants research; therefore, approval was waived. The sample was limited to hospitals with financial data deemed comparable to other hospitals by the California Office of Statewide Health Planning and Development. Noncomparable hospitals excluded from the analysis included Kaiser Permanente hospitals that report consolidated data for many hospitals, psychiatric hospitals, long-term care hospitals, Shriners hospitals that do not charge for care, and state hospitals that provide care for patients with mental and developmental disabilities. The analysis sample included 3480 observations in a panel of 348 hospitals observed for 10 quarters (January 2019 to June 2021). This cross-sectional study followed the Strengthening the Reporting of Observational Studies in Epidemiology (STROBE) reporting guideline.

### Statistical Analysis

For each quarter, we calculated mean operating revenues, operating expenses, net operating income, nonoperating income and expenses, and net income for the sample of 348 hospitals from the first quarter of 2019 to the second quarter of 2021.

Total revenue was calculated by summing inpatient revenue, outpatient revenue, and other operating revenue. Other operating revenue included various revenues, such as nonpatient food sales, sale of drugs to individuals who were not patients, medical records, parking, and tuition from medical and nursing schools. In addition, the Centers for Medicare & Medicaid Services requires hospitals to report the PRF payments as other operating revenue on the Medicare Cost Report.^15^ However, it is not possible to directly measure the amount of assistance because The Hospital Quarterly Financial and Utilization Data contains a single field for all items belonging to other operating revenue and does not break out subcategories.

Our results focus on 2 forms of profitability: net operating income and net income. Net operating income is a component of net income and represents profit after total operating expense is deducted from total revenue, but before nonoperating income and expense is considered. Thus, we calculated net operating income as the total of inpatient, outpatient, and other operating revenue less expenses from patient care. After calculating net operating income, nonoperating revenues less expenses were subtracted to reach net income. These nonoperating revenues less nonoperating expenses included revenue from real estate rental income, rental and medical building expenses, unrestricted donations, governmental appropriations, housing expenses, retail operations expenses, and investment income. The data did not break out categories of this variable separately, but other research found that the largest category of nonoperating income less nonoperating expenses was investment income.^16^ For each hospital, we calculate operating margin by dividing net operating income by total revenue, and profit margin by dividing net income by total revenue.

Next, we subset our sample of 348 hospitals into 247 non-SNHs and 101 SNHs and analyzed the financial performance of them. Centers for Medicare & Medicaid Services has traditionally used the Medicare Disproportionate Share Hospital index to define SNHs.^17^ Furthermore, the eligibility criteria for the PRF Safety Net Hospitals Targeted Distribution required hospitals to have a Medicare Disproportionate Patient Percentage of 20.2% or higher.^18^ We followed this definition and hospitals were classified as an SNH if Medicaid and indigent discharges as a percentage of total discharges were within the top quartile in 2019.

The first detected COVID-19 case in the US was confirmed by the Centers for Disease Control and Prevention on January 21, 2020, and COVID-19 was declared a national emergency on March 13, 2020.^19^ Therefore, the 4 quarters of 2019 were considered pre–COVID-19 periods and all subsequent quarters were COVID-19 periods.

We calculated sample means and 95% CIs to compare groups. For primary analysis, statistical significance was not assessed. All dollar values were deflated to first quarter 2019 prices using the hospital price index.^20^ When statistical tests were performed, statistical significance was set at 2-sided *P* < .05. Data analyses were performed using Stata version 15.0 (Stata Corp).

## Results

### Overall Financial Performance

Table 1 presents mean revenues, expenses, and income for the 348 hospitals from the first quarter of 2019 to the second quarter of 2021. Mean net operating income was the lowest in second quarter 2020 at −$2.7 million (95% CI, −$6.5 million to $1.1 million). Despite operating losses, large increases in other operating revenue (mean, $9.3 million; 95% CI, $7.1 million to $11.4 million) and nonoperating revenue (mean, $4.3 million; 95% CI, $1.7 million to $6.8 million) mitigated losses as government assistance programs and the stock market recovery took effect during the quarter. Mean net operating income was again negative in the first quarter of 2021 (mean, −$1.1 million; 95% CI, −$3.6 to $1.5 million) and positive during other quarters.

**Table 1.** Revenues, Expenses, and Income for All Hospitals^a^

Variable	Quarter									
	2019				2020				2021	
	First	Second	Third	Fourth	First	Second	Third	Fourth	First	Second
Revenue in US $ millions										
Total	83.053	87.468	80.818	81.129	84.257	78.810	82.567	84.863	83.403	86.423
Inpatient	49.033	50.215	45.934	46.444	49.437	44.217	46.999	48.956	49.489	47.768
Outpatient	30.768	33.509	31.351	31.189	31.463	25.311	29.949	30.636	29.389	34.335
Other operating	3.252	3.744	3.532	3.496	3.357	9.281	5.619	5.272	4.525	4.321
Total operating expenses	78.481	80.943	79.698	80.713	83.424	81.488	81.153	84.490	84.478	82.686
Inpatient	48.163	48.485	47.402	48.257	50.865	51.905	49.681	52.130	52.935	48.346
Outpatient	30.318	32.458	32.297	32.456	32.559	29.583	31.472	32.360	31.543	34.340
Net operating income	4.573	6.525	1.120	0.415	0.832	−2.678	1.413	0.373	−1.075	3.737
Nonoperating revenue less nonoperating expenses	4.137	2.986	1.765	7.574	−2.636	4.270	5.436	7.687	4.832	6.466
Net income	8.709	9.511	2.885	7.989	−1.804	1.592	6.849	8.060	3.756	10.203
Margin, %										
Operating	2.9	5.1	−0.9	−0.8	−1.2	−3.6	0.9	0.3	−1.2	2.3
Profit	7.1	8.4	1.2	7.7	−1.9	1.7	5.5	6.5	2.2	7.5
No. of hospitals	348	348	348	348	348	348	348	348	348	348

^a^ Table shows mean revenue, expenses, and margins for 348 California hospitals between the first quarter of 2019 and the second quarter of 2021, shown in millions of dollars. Data are the Hospital Quarterly Financial and Utilization Data from the State of California Office of Statewide Health Planning and Development. The analysis sample included 3480 observations in a panel of 348 hospitals observed for 10 quarters.

Mean net income was the lowest during the first quarter of the pandemic in the first quarter of 2020 at −$1.8 million (95% CI, −$4.2 million to $0.5 million). This decrease in net income is associated with nonoperating revenue less nonoperating expenses which decreased by a mean of $10.2 million from $7.6 million (95% CI, $5.2 million to $9.9 million) in the fourth quarter of 2019 to −$2.6 million in the first quarter of 2020 (95% CI, −$5.0 million to −$0.2 million). All subsequent quarters mean net income was positive and comparable to prepandemic levels.

**Figure 1.**
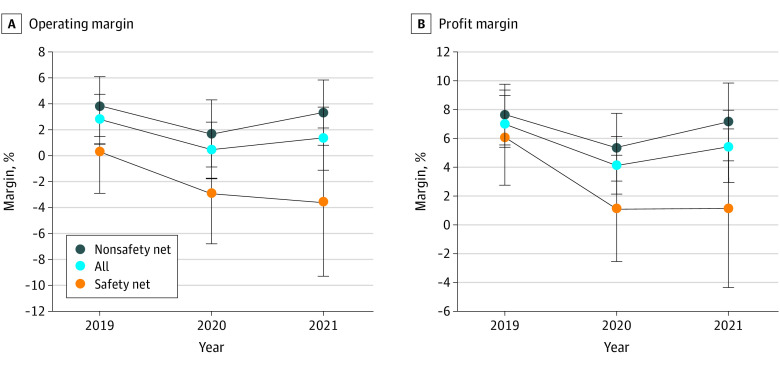
Annualized Operating Margin and Profit Margin From Q1 2019 to Q2 2021 Data are the Hospital Quarterly Financial and Utilization Data from the State of California Office of Statewide Health Planning and Development and COVID-19. The sample contains 348 hospitals. Q indicates quarter.

For all hospitals, the operating margin decreased from 2.8% (95% CI, 0.9% to 4.7%) in 2019 to 0.4% (95% CI, −1.8% to 2.6%) in 2020 then increased to 1.3% (95% CI, −1.1% to 3.7%) in the first 6 months of 2021 (Figure 1A). Non-SNH hospitals followed a similar pattern. In contrast to non-SNHs, SNH margins did not show a recovery in 2021 and were negative in 2020 and 2021. A similar pattern occurs for total margin (Figure 1B).

**Figure 2.**
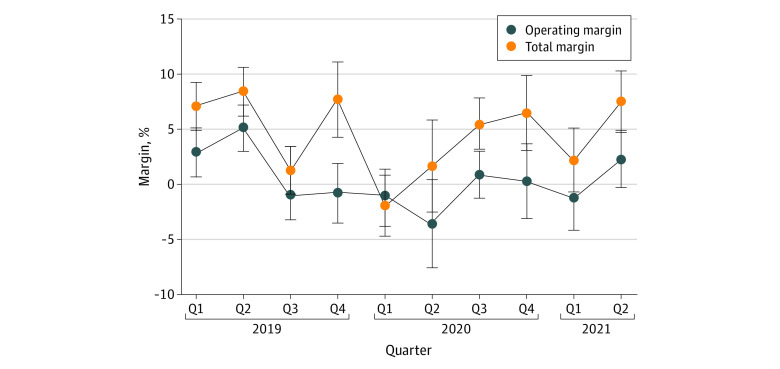
Quarterly Operating Margin and Profit Margin for All Hospitals Q1 2019 to Q2 2021 Data are the Hospital Quarterly Financial and Utilization Data from the State of California Office of Statewide Health Planning and Development and COVID-19. The sample contains 348 hospitals. Q indicates quarter.

Figure 2 presents quarterly operating margin and total profit margin for all hospitals with the same data shown in Table 1. Profit margin decreased substantially to −1.9% (95% CI, −5.0% to 1.0%) in the first quarter of 2020. Operating margin decreased in the second quarter of 2020 but recovered during the subsequent quarters.

### Financial Performance by Safety-Net Status

**Figure 3.**
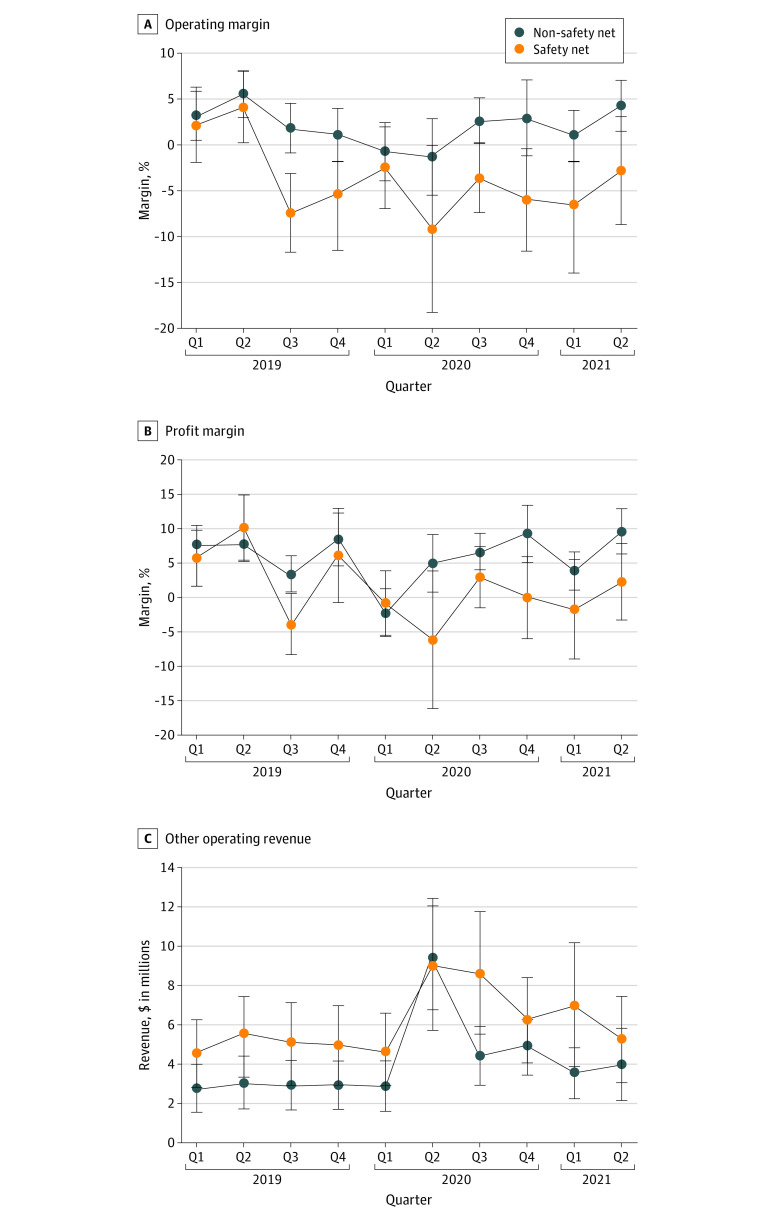
Operating Margin, Profit Margin, and Other Operating Revenue Data Operating margin, profit margin, and other operating revenue data are the hospital quarterly financial and utilization data from the State of California Office of Statewide Health Planning and Development and COVID-19. The sample contains 101 safety-net hospitals and 247 non–safety net hospitals. A safety-net hospital is defined by being in the top quartile of Medicaid and indigent discharges as a percentage of total discharges in 2019.

Non-SNHs fared substantially better than SNHs during the pandemic (Table 2 and Figure 3). For non-SNHs, operating margin was negative only in the first and second quarters of 2020 and quickly returned to positive levels comparable to pre–COVID-19 quarters. In comparison, operating margin for SNHs decreased substantially from the first quarter of 2020 to the second quarter of 2020 and remained lower than pre–COVID-19 quarters for the sample periods (Figure 3A). Similar trends occurred with respect to profit margin (Figure 3B). Between the first quarter of 2020 and the second quarter of 2021, California safety-net hospitals’ net operating losses were more than $3.2 billion. During the course of our research, we found that California SNHs were more likely to be teaching hospitals (16% [170 of 1010 hospital-quarters] vs 5% [120 of 2470 hospital-quarters]; *P*<.001), less likely to be rural (10% [100 of 1010 hospital-quarters] vs 16% [394 of 2470 hospital-quarters]; *P*<.001), have more beds (mean [SD], 248 [18.9] for SNH vs 206 [11.4] for non-SNH; *P*<.001), and were located in areas with lower median household income ($71 709 vs $74 517; *P*<.001).

**Table 2.** Revenues, Expenses, and Income by Safety-Net Status^a^

Variable	Quarter									
	2019				2020				2021	
	First	Second	Third	Fourth	First	Second	Third	Fourth	First	Second
**Non–safety net hospitals**										
Revenue in US $ millions										
Total	82.297	86.374	82.644	82.649	84.049	78.371	83.453	86.483	83.784	88.105
Inpatient	47.822	48.63	46.148	46.403	47.896	41.892	46.477	48.274	48.348	47.284
Outpatient	31.733	34.722	33.595	33.351	33.307	27.1	32.57	33.315	31.919	36.874
Other operating	2.741	3.022	2.901	2.895	2.846	9.379	4.406	4.893	3.518	3.948
Total operating expenses	77.737	79.344	78.644	80.431	82.205	77.829	79.894	83.665	82.276	81.448
Inpatient	46.67	46.272	45.38	46.658	48.462	47.148	46.932	49.541	49.543	45.849
Outpatient	31.067	33.073	33.263	33.773	33.743	30.681	32.962	34.125	32.733	35.599
Net operating income	4.56	7.03	4	2.218	1.844	0.542	3.559	2.817	1.508	6.657
Nonoperating revenue less nonoperating expenses	3.735	1.944	1.003	6.794	−3.628	4.948	4.783	7.691	3.83	5.342
Net income	8.295	8.973	5.003	9.012	−1.784	5.491	8.342	10.508	5.338	11.999
Margin, %										
Operating	3.2	5.5	1.8	1.1	−0.7	−1.3	2.7	2.9	1	4.3
Profit	2.6	2.4	2.6	3.8	3.5	4.2	2.7	4.1	2.8	3.3
No. of hospitals	247	247	247	247	247	247	247	247	247	247
**Safety-net hospitals**										
Revenue in US $ millions										
Total	84.904	90.143	76.353	77.411	84.764	79.882	80.399	80.904	82.469	82.312
Inpatient	51.996	54.09	45.412	46.544	53.204	49.902	48.276	50.624	52.28	48.953
Outpatient	28.408	30.542	25.865	25.9	26.954	20.938	23.539	24.082	23.203	28.125
Other operating	4.5	5.511	5.076	4.966	4.606	9.043	8.584	6.198	6.986	5.234
Total operating expenses	80.301	84.853	82.278	81.403	86.406	90.437	84.233	86.506	89.863	85.715
Inpatient	51.813	53.899	52.344	52.169	56.743	63.54	56.405	58.462	61.231	54.452
Outpatient	28.487	30.954	29.934	29.234	29.663	26.897	27.828	28.044	28.631	31.263
Net operating income	4.603	5.29	−5.925	−3.992	−1.642	−10.554	−3.834	−5.603	−7.393	−3.403
Nonoperating revenue less nonoperating expenses	5.118	5.535	3.629	9.481	−0.21	2.61	7.032	7.678	7.282	9.212
Net income	9.721	10.825	−2.296	5.488	−1.852	−7.944	3.198	2.075	−0.111	5.809
Margin, %										
Operating	2.2	4.2	−7.4	−5.4	−2.5	−9.2	−3.6	−6	−6.5	−2.8
Profit	4.1	4.8	4.4	6.9	4.8	10	4.5	6	7.2	5.6
No. of hospitals	101	101	101	101	101	101	101	101	101	101

^a^ Table shows mean revenue, expenses, and margins for 247 non–safety net hospitals and 101 safety-net hospitals between the first quarter of 2019 and the second quarter of 2021, in millions of dollars. Data are the Hospital Quarterly Financial and Utilization Data from the State of California Office of Statewide Health Planning and Development. The analysis sample included 3480 observations in a panel of 348 hospitals observed for 10 quarters.

The PRF played an important role in the finances of both SNH and non-SNHs (Figure 3C). From the first quarter of 2019 to the first quarter of 2020, other operating revenue stayed relatively constant for both SNHs and non-SNHs. Increases in other operating revenue were observed in the second quarter of 2020, from $4.6 million (9.5% CI, $2.8 million to $6.5 million) to $9.0 million (95% CI, $5.6 million to $12.4 million) for SNHs. For non-SNHs, other operating revenue increased from $2.8 (95% CI, $1.6 million to $4.1 million) to $9.4 million (95% CI, $6.7 million to $12.0 million). Although subsequent quarters experienced reductions relative to the second quarter of 2020, other operating revenue stayed higher than prepandemic levels. The decrease in other operating revenue was smaller for SNHs, likely representing additional PRF targeted at SNHs.

## Discussion

In this cross-sectional study, we examined the financial performance of California hospitals during the first 6 COVID-19 quarters. The first 2 pandemic quarters (the first quarter of 2020 and second quarter of 2020) were characterized by reduced net operating income and net income. Net profitability of hospitals was associated with fluctuations in other operating revenue and nonoperating revenue less nonoperating expenses. Government assistance played an important role in the increase in other operating revenue and helped to contain what could have been a much more dire situation for hospitals. In addition, the fluctuations in nonoperating revenue less expense is associated with financial market performance, which further helped bolster hospital profits from the second quarter of 2020 to the second quarter of 2021.^21^ These trends in profitability were not evenly distributed across all hospitals because changes were more pronounced among SNHs.

### Limitations

This study has limitations. First, the data are limited to the state of California, which may not be generalizable to the US. Nevertheless, California comprises 12% of the US population and has had the most COVID-19 cases and deaths to date.^22^ Studying California is worthwhile because the California Hospital Quarterly Financial and Utilization Data are quarterly whereas other hospital financial data available at the annual level (eg, Centers for Medicare & Medicaid Services hospital cost reports, American Hospital Association Annual Survey) lack the granularity to measure within-year dynamics in financial performance. As we have reported, net income was variable with periods of substantial decreases and increases relative to the pre–COVID-19 period.

The second limitation of this study is that COVID-19 relief payments and changes in the value of equity market holdings are grouped into variables with other types of income and expenses. This data limitation prevents us from quantifying the exact contribution of these items to changes in profitability. However, the goal of this article is to track overall changes in hospital profits during the pandemic. The allocation of COVID-19 PRF^23,24^ and nonoperating income’s role in hospital profitability during the prepandemic period has been studied elsewhere.^16,25^ Future research should use different data to explore these items in terms of profit outcomes.

## Conclusions

In this cross-sectional study of California hospitals, we found a negative association between COVID-19 and hospital financial performance. Although hospitals experienced reduced profits between January 2020 and June 2021, the intervention of government assistance programs was able to mitigate more detrimental fiscal consequences. When compared with non-SNHs, SNHs had lower profits and received more government assistance.

## Notes

Supplement. **eFigure.** Non-Operating Income Less Non-Operating Expenses and S&P 500 Performance
