# Supplementary material for: Financial Outcomes Associated With the COVID-19 Pandemic in California Hospitals
Source: JAMA Health Forum. 2022 Sep 23;3(9):e223056. doi: 10.1001/jamahealthforum.2022.3056 (PMC9508652; doi:10.1001/jamahealthforum.2022.3056)
Supplement: Supplement. — eFigure. Non-Operating Income Less Non-Operating Expenses and S&P 500 Performance [file jamahealthforum-e223056-s001.pdf]

Supplemental Online Content

Wang Y, Witman AE, Cho DD, Watson ED. Financial outcomes associated with the COVID-19 pandemic in California hospitals. *JAMA Health Forum.* 2022;3(9):e223056. doi:10.1001/jamahealthforum.2022.3056

eFigure. Non-Operating Income Less Non-Operating Expenses and S&P 500 Performance

This supplemental material has been provided by the authors to give readers additional information about their work.

© 2022 Wang Y et al. *JAMA Health Forum*.

eFigure: Non-Operating Income Less Non-Operating Expenses and S&P 500 Performance

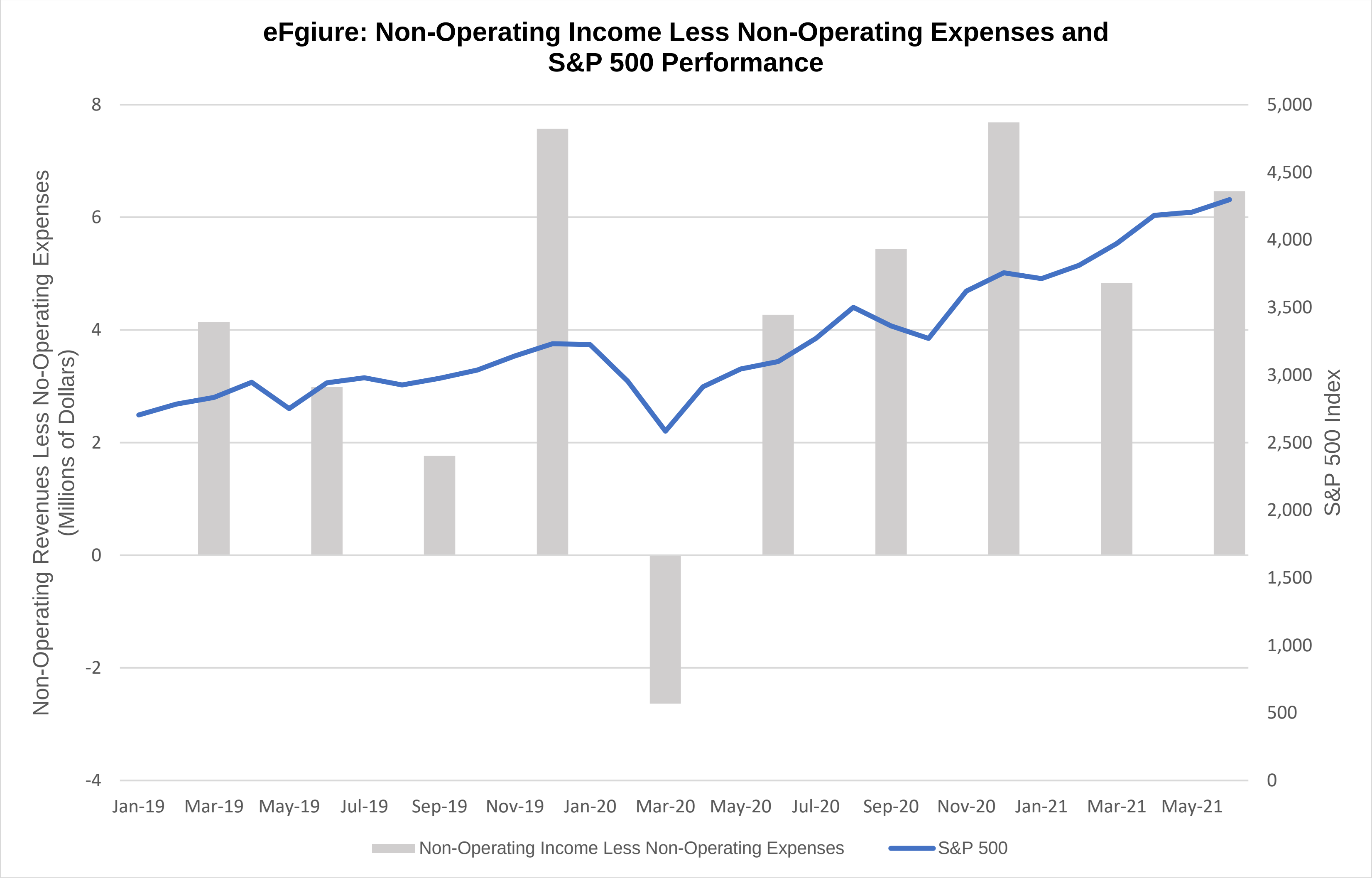

© 2022 Wang Y et al. *JAMA Health Forum*.
